# Editorial: Integration of next-generation technologies with biosensors for advanced diagnostics and personalized medicine

**DOI:** 10.3389/fbioe.2026.1963463

**Published:** 2026-08-26

**Authors:** Gabriel Avelino Sampedro, Made Adi Paramartha Putra, Dong-Seong Kim

**Affiliations:** 1 College of Technology, Lyceum of the Philippines University, Intramuros, Philippines; 2 Department of IT Convergence Engineering, Kumoh National Institute of Technology, Gumi, Gyeongbuk, Republic of Korea; 3 Faculty of Technology Information and Design, Primakara University, Kota Denpasar, Bali, Indonesia

**Keywords:** artificial intelligence, biosensors, digital health, nanotechnology, point-of-care diagnostics, wearable devices

## 1 Introduction

The convergence of biosensors with artificial intelligence (AI), advanced materials, miniaturized electronics, microfluidics, wireless communication, and computational modeling is reshaping diagnostics and personalized medicine. Biosensors are increasingly moving beyond isolated laboratory measurements toward integrated systems that acquire physiological or biochemical signals, interpret them in real time, and support decisions tailored to individual patients. This transition is particularly important where conventional assessment remains invasive, episodic, costly, or unable to capture rapidly changing physiological states.

The Research Topic “Integration of Next-Generation Technologies with Biosensors for Advanced Diagnostics and Personalized Medicine” brings together 14 articles that illustrate this broader transformation. The contributions span intelligent biosignal analysis, decentralized testing, nanotechnology, wearable and implantable systems, digital twins, and participatory design. Taken together, they describe a sensing-to-decision continuum in which biological signals are acquired through increasingly sensitive and minimally invasive interfaces, converted into clinically meaningful information, and applied to screening, diagnosis, monitoring, treatment planning, and intervention. The Research Topic emphasizes not only the development of more capable sensors, but also the integration of reliable measurement with trustworthy computation, clinical relevance, and user-centered implementation.

## 2 Intelligent biosensing and decentralized diagnostics

The first group of contributions demonstrates how computational methods can increase the clinical value of physiological, imaging, and biochemical data. Hu et al. combined single-lead electrocardiography, signal decomposition, time–frequency analysis, and random forest classification for sleep apnea detection. Their work shows how a relatively simple and accessible biosignal can support screening when paired with carefully selected spectral features. Extending this direction, Tang et al. proposed a multimodal anomaly-detection framework that integrates symbolic clinical representations, temporally evolving graphs, and medical knowledge. The study highlights the value of combining heterogeneous data while maintaining interpretability and robustness.

The importance of cross-domain learning is further illustrated by Almadhor et al., who examined stress, activity, and emotion recognition across multiple biosignal datasets using deep learning, transfer learning, and temporal modeling. Their results also reveal an important limitation: strong predictive performance does not automatically produce explanations that are meaningful for clinical interpretation. In medical imaging, Xu et al. developed a Swin UNETR model for detecting and measuring alveolar bone fenestration and dehiscence in cone-beam computed tomography. This work demonstrates how AI can support more standardized, quantitative, and personalized orthodontic risk assessment. Najjar et al. combined localized bioimpedance, anthropometric measurements, and machine learning to estimate hand grip strength, showing that size-normalized forearm bioimpedance adds physiologically relevant information beyond conventional body measurements and may support future wearable monitoring of muscle function and rehabilitation.

Other contributions focus on making biochemical analysis more portable and accessible. Li et al. established a quantitative absorptive microsampling and mass-spectrometry method for decentralized monitoring of insulin-like growth factor 1. By reducing sample requirements and supporting decentralized Research Topic, the approach may facilitate longitudinal endocrine monitoring in settings where repeated venous sampling or centralized testing is difficult. Caya et al. developed a compact CMOS potentiostatic readout circuit and portable urinary sensing platform for uric acid, with additional capability for pH-, calcium-, and conductivity-related measurements relevant to urolithiasis monitoring.

At the level of sample transport and preparation, Ciarrocchi et al. addressed the physiological delay between blood glucose and interstitial glucose. Using a microfluidic model, they demonstrated that electro-osmotic transport can reduce diffusion lag, suggesting a route toward more responsive continuous glucose monitoring. Lan et al. reviewed magnetic bead-assisted platforms for extracellular-vesicle isolation, including surface modification, microfluidic integration, and multiplex capture. Their analysis highlights both the promise of efficient biomarker enrichment and the need for standardized isolation and characterization protocols. Together, these studies show that decentralization requires more than miniaturization. Reliable point-of-care and remote systems also depend on reproducible sampling, stable bioelectronic interfaces, calibration, streamlined workflows, and analytical performance that remains dependable outside controlled laboratories.

## 3 Continuous monitoring, smart systems, and clinical translation

A second group of contributions shows how biosensors are evolving from devices that generate isolated measurements into systems capable of continuous observation, dynamic modeling, and, eventually, closed-loop intervention. At the nanoscale, Yu et al. reviewed multifunctional nanoplatforms for early lung-cancer detection and immune modulation. By combining molecular recognition, signal amplification, targeted delivery, and tumor-microenvironment reprogramming, these platforms reflect the increasing convergence of diagnosis and therapy. Their translation, however, will depend on overcoming biological barriers and establishing biocompatibility, manufacturing consistency, scalability, and regulatory safety.

Continuous organ-specific monitoring is represented by Cho et al., who developed flexible gold-coated carbon-nanotube strain sensors for bladder-volume measurement and used their outputs to construct a preliminary “Virtual Bladder.” The study provides a foundation for future digital-twin models and closed-loop neuromodulation for bladder dysfunction. In orthopedics, Wu et al. reviewed sensor-integrated hip and knee prostheses capable of monitoring joint loading, motion, gait-related variables, and local temperature. Such systems may support longitudinal orthopedic phenotyping and earlier identification of complications, although challenges involving long-term power, telemetry, durability, data security, and clinically actionable thresholds remain.

The importance of real-time interpretation in time-critical care is demonstrated by Ortiz et al., who implemented compensatory reserve measurement from wearable photoplethysmographic data during simulated hypovolemia. Their system identified deteriorating physiological trends earlier than conventional vital signs, illustrating how continuous biosensing and computational interpretation may improve hemorrhage triage and intervention.

Technical capability alone, however, does not guarantee meaningful adoption. Quidato et al. reviewed participatory-design studies involving older adults and identified clarity, usability, engagement, and trustworthiness as recurring requirements for miniaturized biosensor-enabled technologies. Their contribution emphasizes that intended users should participate throughout development, particularly when changes in vision, mobility, cognition, privacy expectations, and digital literacy influence sustained use.

Taken together, the articles in this Research Topic reveal several shared priorities. Promising proof-of-concept systems must progress toward larger and externally validated studies involving diverse populations and real-world conditions. Standardization is needed because sensor placement, sampling, fabrication, preprocessing, calibration, dataset composition, and evaluation metrics can substantially affect reported performance. AI-enabled systems must demonstrate not only accuracy, but also robustness, calibration, uncertainty awareness, explainability, and resilience to distribution shifts. Interoperability with electronic health records, decision-support systems, and remote-care workflows is likewise necessary so that continuous data lead to timely action rather than alarm burden or information overload.

The Research Topic shows that next-generation biosensors are becoming central components of intelligent health ecosystems. Across sleep medicine, mental health, oncology, endocrinology, dentistry, orthopedics, rehabilitation, trauma care, diabetes, urology, and aging, the contributions share a common objective: generating health information that is earlier, more continuous, more accessible, and more responsive to individual needs. Progress toward personalized medicine will therefore depend on integrating reliable sensing with transparent computation, inclusive design, rigorous clinical validation, and decisions that are genuinely actionable for patients and healthcare professionals.

